# Invasion/chemotaxis- and extravasation-chip models for breast cancer bone metastasis

**DOI:** 10.1371/journal.pone.0309285

**Published:** 2024-10-17

**Authors:** Burcu Firatligil-Yildirir, Gizem Bati-Ayaz, Nonappa, Devrim Pesen-Okvur, Ozden Yalcin-Ozuysal

**Affiliations:** 1 Department of Molecular Biology and Genetics, Izmir Institute of Technology, Izmir, Turkiye; 2 Izmir Institute of Technology, Biotechnology and Bioengineering Graduate Program, Izmir, Turkiye; 3 Faculty of Engineering and Natural Sciences, Tampere University, Tampere, Finland; Università degli Studi della Campania, ITALY

## Abstract

Bone is one of the most frequently targeted organs in metastatic cancers including the breast. Breast cancer bone metastasis often results in devastating outcomes as limited treatment options are currently available. Therefore, innovative methods are needed to provide earlier detection and thus better treatment and prognosis. Here, we present a new approach to model bone-like microenvironments to detect invasion and extravasation of breast cancer cells using invasion/chemotaxis (IC-) and extravasation (EX-) chips, respectively. Our results show that the behaviors of MDA-MB-231 breast cancer cells on IC- and EX-chip models correlate with their *in vivo* metastatic potential. Our culture model constitutes cell lines representing osteoblasts, bone marrow stromal cells, and monocytes embedded in three-dimensional (3D) collagen I-based extracellular matrices of varying composition and stiffness. We show that collagen I offers a better bone-like environment for bone cells and matrix composition and stiffness regulate the invasion of breast cancer cells. Using *in situ* contactless rheological measurements under cell culture conditions, we show that the presence of cells increased the stiffness values of the matrices up to 1200 Pa when monitored for five days. This suggests that the cellular composition has a significant effect on regulating matrix mechanical properties, which in turn contribute to the invasiveness. The platforms we present here enable the investigation of the underlying molecular mechanisms in breast cancer bone metastasis and provide the groundwork of developing preclinical tools for the prediction of bone metastasis risk.

## Introduction

Breast cancer is the most frequently diagnosed cancer in women, and the majority of breast cancer-related deaths occur due to metastasis [1]. The most common sites of breast cancer metastasis include bone, lung, liver, and brain [2]. The metastasis risk is mainly associated with the stage of the tumor at the time of diagnosis, as well as histological and molecular subtypes of breast cancer. However, diagnostic tools to predict the metastatic potential and/or the target organ identity, which will improve the accuracy of diagnosis and prognosis as well as treatment planning, are not currently available in the clinic.

Bone is one of the most frequently targeted organs in metastatic cancers, including the breast. Recent studies estimate the bone metastasis rate as 53.71% in metastatic breast cancer [3]. In a 10-year follow-up period, bone metastasis incidence reaches 61% for the patients diagnosed with stage IV breast cancer [4]. Bone metastases are devastating for patients as they often result in severe symptoms such as pain, pathological bone fractures, and spinal cord compressions, increasing the need for better diagnostic and prognostic tools as well as novel treatment strategies [5]. Thus, it is crucial to establish physiologically relevant models for investigating cellular and molecular aspects of bone metastasis.

In recent years, lab-on-chip (LOC) platforms have emerged as powerful tools in cancer research, allowing the generation of physiologically relevant disease and diagnostic models. LOC systems offer accurate design of a wide range of architectures and compartments for fine control of mechanical properties and physiological parameters to accommodate different cell types in three-dimensional (3D) microenvironments [6]. Several lab-on-chip platforms have been developed to model bone microenvironment and study bone-related functions and metastasis [7]. Torisawa et al. reported a bone marrow-on-a-chip platform to combine *in vitro-*generated bone and *in vivo-*produced bone marrow [8]. The *in vivo* bone engineering allowed the population of an artificial bone extracellular matrix with mouse hematopoietic stem and progenitor cells, which survived for up to one week *in vitro* and served as a platform to analyze radiation toxicity [8]. Other approaches not requiring *in vivo* transplantation exploited osteoblast or osteocyte cell lines, [9–12] or mesenchymal stem cells (MSCs) derived from bone marrow [13–15] or embryonic stem cells [16]. To mimic physiologically relevant bone microenvironment, MSCs differentiated under osteogenic conditions, [13, 14, 16] and endothelial cells were added to the culture [11, 13–15, 17]. These bone-on-chip platforms led to the *in vitro* visualization of extravasation, [13, 14] invasion [9, 11] and colonization [10, 15]. Furthermore, it also allowed the investigation of molecular mechanisms involved in extravasation such as CXCL5-CXCR2 interaction, [14] and the role of proteases in bone metastasis [18]. However, a more practical approach, which does not require an *in vivo* transplantation or prolonged differentiation steps yet represents the complexity of the bone microenvironment, remains to be established.

Recently, we developed two LOC platforms, invasion/chemotaxis (IC)-chip and extravasation (EX)-chip, to determine the tissue-specific metastatic potential of breast cancer cells [19]. The IC- and EX-chips allowed visualization and quantification of invasion and extravasation potentials of metastatic breast cancer cell line MDA-MB-231 towards lung, liver, and breast microenvironments. Furthermore, we tested the platforms with MDA-MB-231 clones LM2 and BoM 1833, which specifically metastasize to lung and bone, respectively, in mouse models [20–22]. LM2 cells invaded and extravasated into the lung microenvironment in the LOC platforms more efficiently than the BoM 1833 cells, reflecting their *in vivo* metastatic preferences.

Here, we utilize the IC- and EX-chip platforms to model a novel bone-like cellular microenvironment comprising cell lines representing osteoblasts, stromal cells, and monocytes embedded in 3D collagen I-based extracellular matrices. We show that the invasion and extravasation potentials of the MDA-MB-231 clones towards this microenvironment on IC- and EX-chips correlate with their *in vivo* behaviors. Our platforms enable a straightforward approach to providing *in vitro* data representative of the *in vivo* bone metastasis risk for breast cancer cell lines. Further improvement of our platforms would allow the analysis of clinical samples and pave the way for developing metastatic site-specific clinical approaches.

## Materials and methods

### Cell lines

Human breast cancer cell lines MDA-MB-231, human normal bone marrow stromal cell line HS5, and human umbilical vein endothelial cell line (HUVEC-C) were obtained from ATCC. Human fetal osteoblast cell line hFOB 1.19, human osteosarcoma bone stromal cell line Saos2, and mouse bone marrow mesenchymal stem cells D1 ORL UVA (D1) were gifts from Engin Özçivici Lab and human histiocytic lymphoma monocytes U937 was a gift from H. Cumhur Tekin Lab at Izmir Institute of Technology. Organ-specific metastatic clones of MDA-MB-231, lung-specific metastatic clone (LM2), and bone-specific metastatic clone (BoM 1833), were gifts from the Joan Massagué Lab in Memorial Sloan Kettering Cancer Center. MDA-MB-231, LM2, BoM 1833, HS5, Saos2, D1, and U937 were cultured in DMEM high glucose (11965092, Gibco) with 10% fetal bovine serum (FBS) (A3840001, Gibco) and 1% Penicillin/Streptomycin (15070063, Gibco). hFOB 1.19 was cultured in DMEM-F12 high glucose (11330057, Gibco) 10% FBS and 1% Penicillin/Streptomycin. HUVEC-C was cultured in DMEM-F12K high glucose (01-095-1A, Biological Industries) with 10% FBS, 0.1 mg/ml Heparin (H3393, Sigma), 0.05mg/mL endothelial cell growth supplement (354006, Sigma) and 1% Penicillin/Streptomycin. All cell lines were cultured at 37°C in a humidified incubator with 5% CO_2_. MDA‐MB‐231, LM2, and BoM 1833 were stably labeled with a red fluorescent protein (DsRed) following a previously reported procedure [19].

### Extracellular matrix and 3D cell culture

Cells were embedded in 4 mg/mL final concentration of Growth Factor Reduced (GFR) Matrigel (GFR-Matrigel) (354230, Corning) as explained previously [19]. Collagen type I (C3867, Sigma) was prepared at 4 or 6 mg/mL concentration with phosphate-buffered saline (PBS) and 1 N NaOH. Then cells prepared in serum-free media were mixed with collagen I solution in a 1:1 ratio to obtain 2 or 3 mg/mL final concentration of collagen I. Agarose (A9539, Sigma) was dissolved in PBS to obtain 20, 10, or 5 mg/mL concentration, heated until completely dissolved, and cooled to around 37°C. Then, the agarose solution is mixed at a 1:1 ratio with the cells in 6 mg/mL of collagen I to obtain a final concentration of 3 mg/ml collagen I and 10, 5, or 2.5 mg/mL of agarose. Chitosan (448877, Sigma) was dissolved in 1% acetic acid solution by overnight stirring at 30 mg/mL concentration and pH was adjusted to 7.2–7.5 with NaOH. Then chitosan solution, 9 mg/mL collagen I solution, and cell mixture were mixed at a 1:1:1 ratio to obtain a final concentration of 10 mg/mL chitosan and 3 mg/mL collagen I.

### Cell viability assay

hFOB, HS5, and U937 cells were embedded into collagen I only (3 mg/mL), collagen I with chitosan (3 mg/mL and 10 mg/mL, respectively), and collagen I with agarose (3 mg/mL and 2.5 mg/mL, respectively). After 3 days of incubation, the viability of cells was determined using ReadyProbes Cell Viability Imaging Kit (R37609, Invitrogen) according to the manufacturer’s instructions. Cells were visualized in 3D using a Leica SP8 confocal microscope and all the cells (blue) and dead cells (green) were counted in three independent experiments. Alive cell numbers were calculated as the difference between the numbers of total and dead cells. The cell viability data is presented as the percentage of viable cells among total cells.

### Invasion and extravasation assays

**Fig 1:**
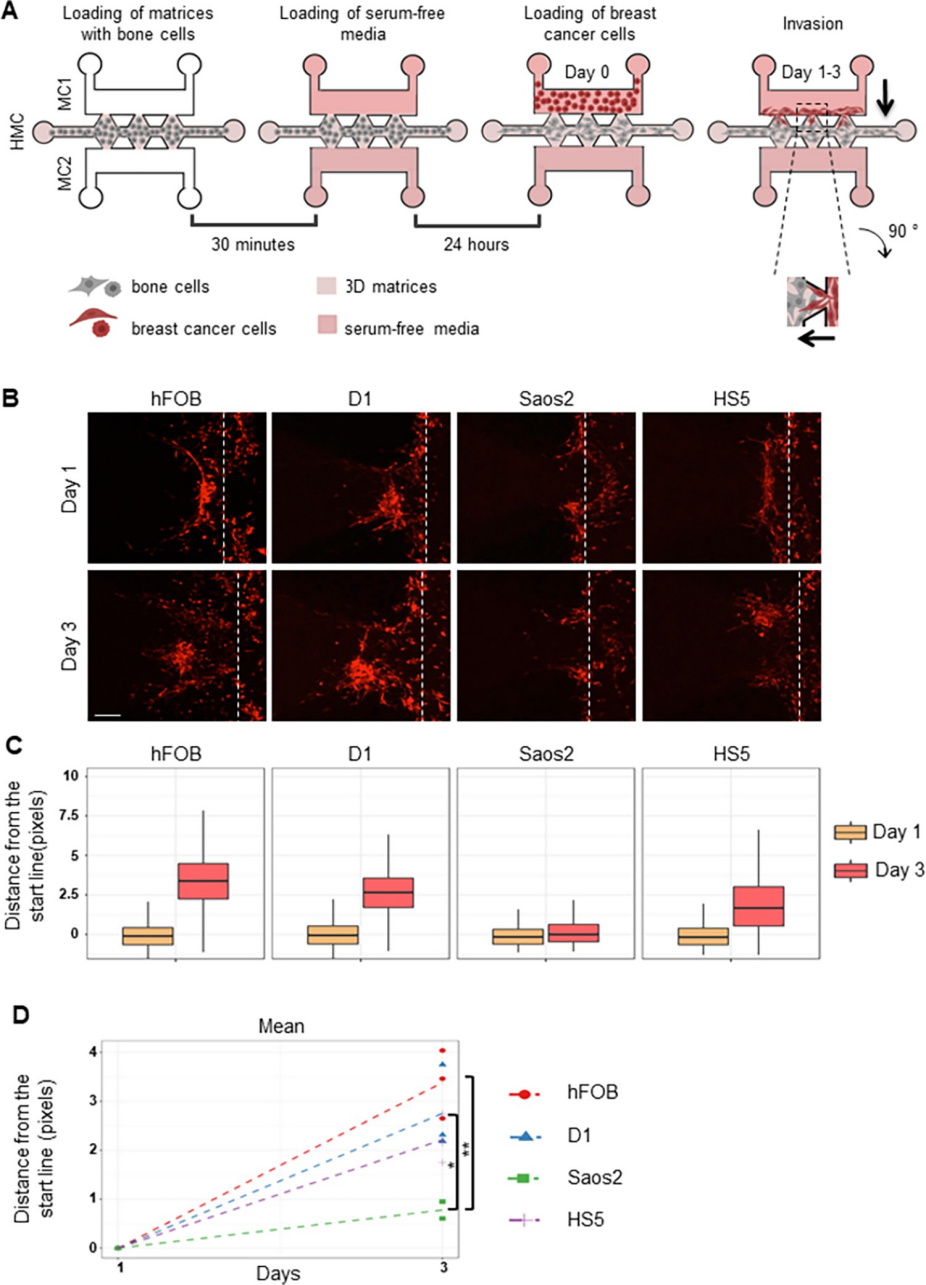
Invasion/chemotaxis of breast cancer cells towards different bone cell types. (A) A schematic representation of the invasion assay on IC-Chip is shown. On the right, a zoom-in inset of the imaging area between two posts is shown. Arrows indicate the direction of invasion. (HMC: Homing Matrix Channel, MC: Medium Channel) (B) Representative Z-stack projection images showing invasion of MDA-MB-231 breast cancer cells (red) towards hFOB (osteoblast), D1 (bone marrow-derived mesenchymal stem cells), Saos2 (osteosarcoma cell line with osteoblastic properties) and HS5 (bone marrow-derived stromal cell) embedded in GFR-Matrigel. The dashed line represents the starting line of invasion/chemotaxis. (Scale bar: 100 μm) The distance of each bright pixel to the starting line was calculated and the data was normalized to Day 1. (C) Distribution and (D) mean of distance values were plotted (n = 3). * p< 0.01, ** p<0.005.

Invasion and extravasation assays were performed on IC-chip and EX-chip, respectively, as explained previously [19]. The chips were provided by Initio Cell Biyoteknoloji (Turkiye). Briefly, the homing matrix channel (HMC) was loaded with individual hFOB, D1, Saos2, or HS5 cell lines at the concentration of 5x10^6^ cells/mL, or in combination at 2x10^6^ cells/mL concentration for each cell line (Figs 1A and 5A). In the IC-chip, serum-free media was added to medium channels (MC) after the polymerization of the hydrogels, and the chips were incubated for a day. In EX-Chip, after polymerization of the hydrogels, the endothelial monolayer channel (EMC) was covered with HUVEC-C cells as described previously and the chips were incubated for 16–24 hours. Then, cancer cells at the concentration of 1x10^6^ cells/mL were loaded to MC or EMC of IC- and EX-Chips, respectively, and visualized for three days using a Leica SP8 confocal microscope. Analyses of invasion and extravasation were done as previously described [19].

### In situ contactless rheological measurements

The storage (elastic) moduli (*G´*) of collagen I-based matrices with or without bone-related cells were measured using the non-destructive *in situ* contactless Elastosens^TM^ Bio (Rheolution, Canada, Montreal) rheometer. In a typical experiment, 250 μL of collagen I only, collagen I + agarose, and collagen I + chitosan with and without cell solutions were transferred to a microvolume holder, and their polymerization was initiated at 37°C. For Day 0, the elastic modulus of the samples was measured immediately after forming the gel within the sample holder. The samples together with the holder were then kept in a 12-well plate having complete cell culture media. The incubation of the samples was carried out for 5 days and each day the elasticity of the samples was measured. The measurements were performed in triplicates for each condition.

## Results and discussion

### Bone-like microenvironment model requires a combination of different bone cell types

To develop a physiologically relevant bone-like microenvironment model, first, we tested how metastatic breast cancer cell line MDA-MB-231 invades towards microenvironments that constitute different bone cell types in a three-channel LOC platform (Fig 1A). Specifically, we used hFOB, D1, Saos2 or HS5 cells to create bone-like microenvironment. hFOB 1.19 human-derived osteoblast cells are commonly used for studies on human osteoblast differentiation and function as they exhibit osteoblast phenotype and express several osteoblastic markers. HS5 cells serve as a model for bone stromal cells, which are essential components of bone marrow and bone pre-metastatic niche, affecting the metastasis of breast cancer cells [23]. HS5 cells are commonly used as a component of the bone-like microenvironment models in various cancers, including breast [24, 25]. D1 ORL UVA (D1), mouse-derived bone marrow mesenchymal stem cells can differentiate into osteocytes and are often utilized for *in vitro* bone tissue engineering [26]. Despite being osteosarcoma cell lines, Saos2 cells display osteoblastic properties. Therefore, they are commonly used in bone tissue engineering applications [27]. In our study, first hFOB, D1, Saos2, or HS5 cells were loaded into homing matrix channel (HMC) of the IC-chip in GFR-Matrigel (Fig 1A). After 24 hours, MDA-MB-231 cells were added into medium channel-1 (MC1) and invasion was observed for three days (Fig 1A). Invasion of MDA-MB-231 cells increased from Day 1 to Day 3 in all conditions, except Saos2 (Fig 1B and 1C). The mean distance invaded by MDA-MB-231 cells was significantly higher towards hFOB and D1 cells compared to Saos2 (Fig 1D).

**Fig 2:**
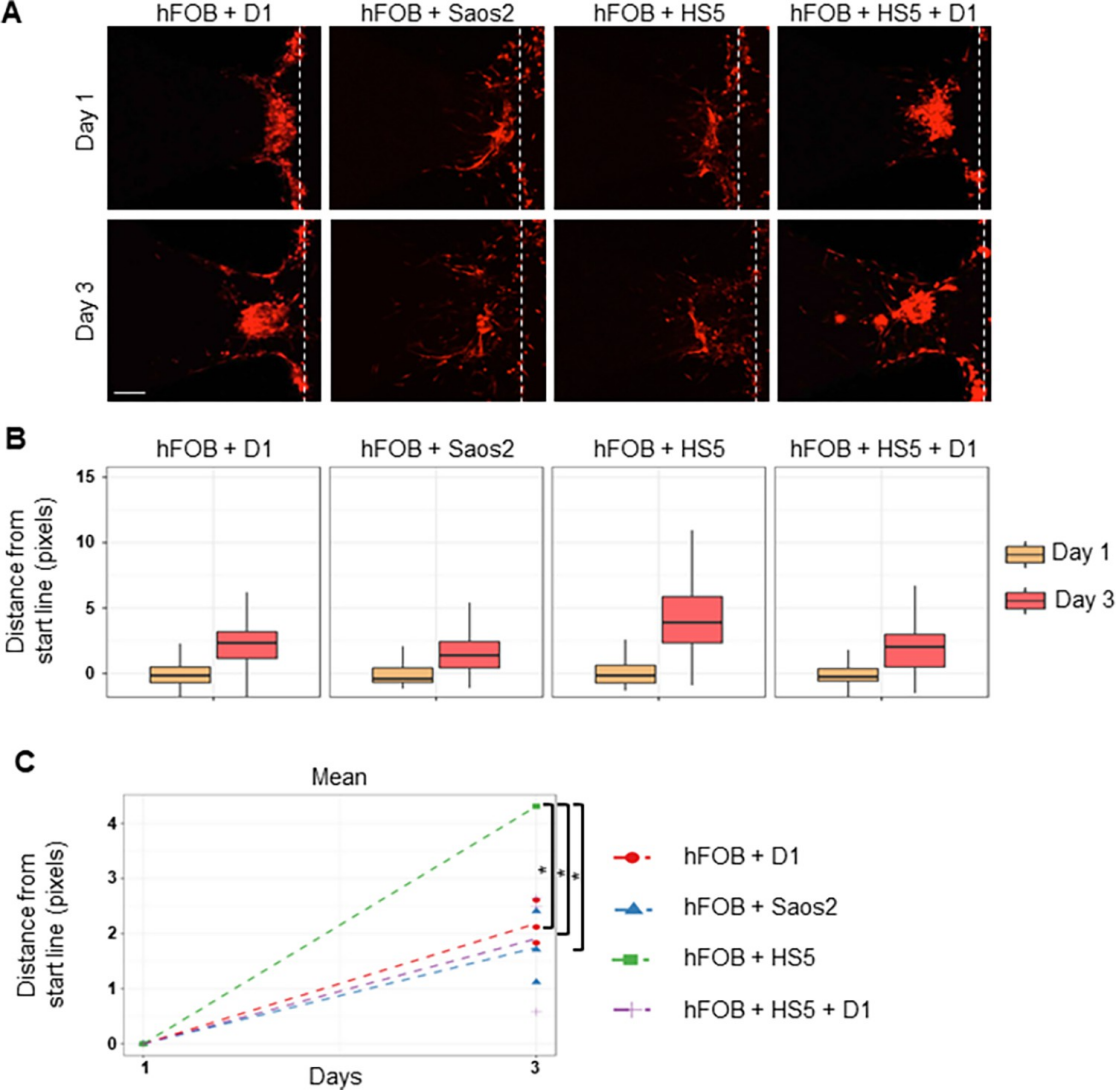
Invasion/chemotaxis of breast cancer cells towards the combination of different bone cell types. (A) Representative Z-stack projection images showing invasion of MDA-MB-231 breast cancer cells (red) towards microenvironments formed by the combination of cell lines embedded in GFR-Matrigel. Dashed lines represent the starting line of invasion/chemotaxis. (Scale bar: 100 μm) The distance of each bright pixel to the starting line was calculated and the data was normalized to Day 1. (B) Distribution and (C) mean of distance values were plotted (n = 3). * p<0.005.

Next, we tested whether invasion increased when hFOB cells were supplemented with other cell types. Accordingly, hFOB cells were seeded in HMC together with D1, Saos2, HS5 or both D1 and HS5. Although the invasion was increased from Day 1 to Day 3 in all conditions, the least distance invaded was towards the environment containing hFOB and Saos2 (Fig 2A and 2B). The longest distance invaded by MDA-MB-231 cells was towards the microenvironment with hFOB and HS5 cells (Fig 2B and 2C). We excluded D1 and Saos2 cells for the subsequent experiments since limited invasion was observed with their presence. Moreover, D1 cells originate from mouse and Saos2 cells are osteosarcoma cells representing a distinct matrix composition by forming apatites and collecting phosphate and calcium compared to normal osteoblasts. Therefore, they might misguide our platform for mimicking native-like bone microenvironments.

Altogether, these results revealed that the invasion of MDA-MB-231 cells was evidently affected by different microenvironments formed by a variety of cell lines originating from the same tissue. Notably, a combination of hFOB osteoblasts with HS5 bone stromal cells provided the most supportive microenvironment for the invasion of breast cancer cells.

### Collagen I offers a better bone-like environment for bone cell types

Matrigel consists of basement membrane components such as laminin, collagen type IV, entactin, and heparan sulfate proteoglycan [28]. Although it is widely used to generate biologically relevant 3D microenvironments, Matrigel does not accurately represent bone matrix. The toughness, tensile stiffness, and elastic properties of the bone microenvironment are mainly supported by the organic matrix, mostly composed of collagen I, which provides adequate mechanical properties to model the bone microenvironment [29]. Collagen I is used as the hydrogel in many *in vitro* applications to mimic bone microenvironment at a concentration ranging between 0.5 mg/mL to 6 mg/mL [30, 31]. Contraction of the hydrogel is a crucial limitation for our platforms since it will lead to the leaking of tumor cells into the homing matrix channel (HMC) without invasion or extravasation. Thus, we first tested 2 mg/mL as the starting concentration, which is within the range of values used in the literature and at the same time shows low contraction [32]. However, the 2 mg/mL collagen I loaded into HMC in the presence of HS5 bone marrow stromal cells contracted and detached from the PDMS borders in two days (S1 Fig). Increasing the concentration of collagen I to 3 mg/mL prevented the contraction (S1 Fig). Previously, it was shown that MDA-MB-231 breast cancer cells have a similar extravasation and migration behavior towards 2 mg/mL and 6 mg/mL collagen I hydrogels in microfluidic platforms, suggesting that the matrix density alone is not a major factor [13, 33]. Thus, we did not test higher concentrations and fixed the collagen I concentration to 3 mg/mL for the following experiments.

### Matrix composition and stiffness affect the invasion of breast cancer cells

The matrix stiffness is one of the strong regulators in various cellular processes, including cell growth, adhesion, migration, invasion, and cell fate, which has been reported in recent studies [34, 35]. The degree of stiffness has important impacts on the diffusion of nutrients among the cells and the activation of intracellular communication through mechanotransduction. Therefore, modulation of matrix stiffness is a parameter to be considered for a more physiologically relevant 3D bone microenvironment. However, tuning the mechanical properties of collagen I has challenges. Therefore, we used chitosan and agarose for the collagen I matrix to prepare a composite bone microenvironment. Chitosan is a widely used cationic polysaccharide in various bone remodeling and regeneration applications [36]. In addition to providing a porous structure, chitosan also supports the adhesion and proliferation of osteoblasts and mesenchymal stem cells [37]. Agarose is another widely used biocompatible but bioinert polymer. Agarose was recently reported to have strain-stiffening properties, suggesting that its mechanical properties are suitable for 3D cell and patient-derived breast cancer tissue explant culture [38, 39]. Thus, we tested whether adding chitosan or agarose could improve our microenvironment model in terms of invasion and extravasation behaviors of cancer cells.

Chitosan concentration in bone microenvironment models varies mainly between 5 mg/mL to 40 mg/mL [37]. In a collagen I-based hydrogel, 10 mg/mL chitosan was shown to provide a porous structure and support the attachment and proliferation of bone marrow stem cells [40]. HS5 cells embedded in 3 mg/mL collagen I only or collagen I supplemented with 10 mg/mL chitosan maintained elongated fibroblast morphology (S2 Fig). Recently, agarose concentrations between 1.6 mg/mL and 10.2 mg/mL have been studied for their strain-stiffening properties [38]. Thus, we tested 2.5 mg/mL, 5 mg/mL, and 10 mg/mL agarose concentrations in combination with 3 mg/mL collagen I. HS5 cells cultured in only collagen I or in collagen I supplemented with 2.5 mg/mL agarose displayed their elongated fibroblast morphology. On the other hand, they appeared rounded and acquired a spherical morphology in collagen I gels with 5 mg/mL and 10 mg/mL agarose, suggesting a loss of cell-matrix attachment (S3 Fig).

**Fig 3:**
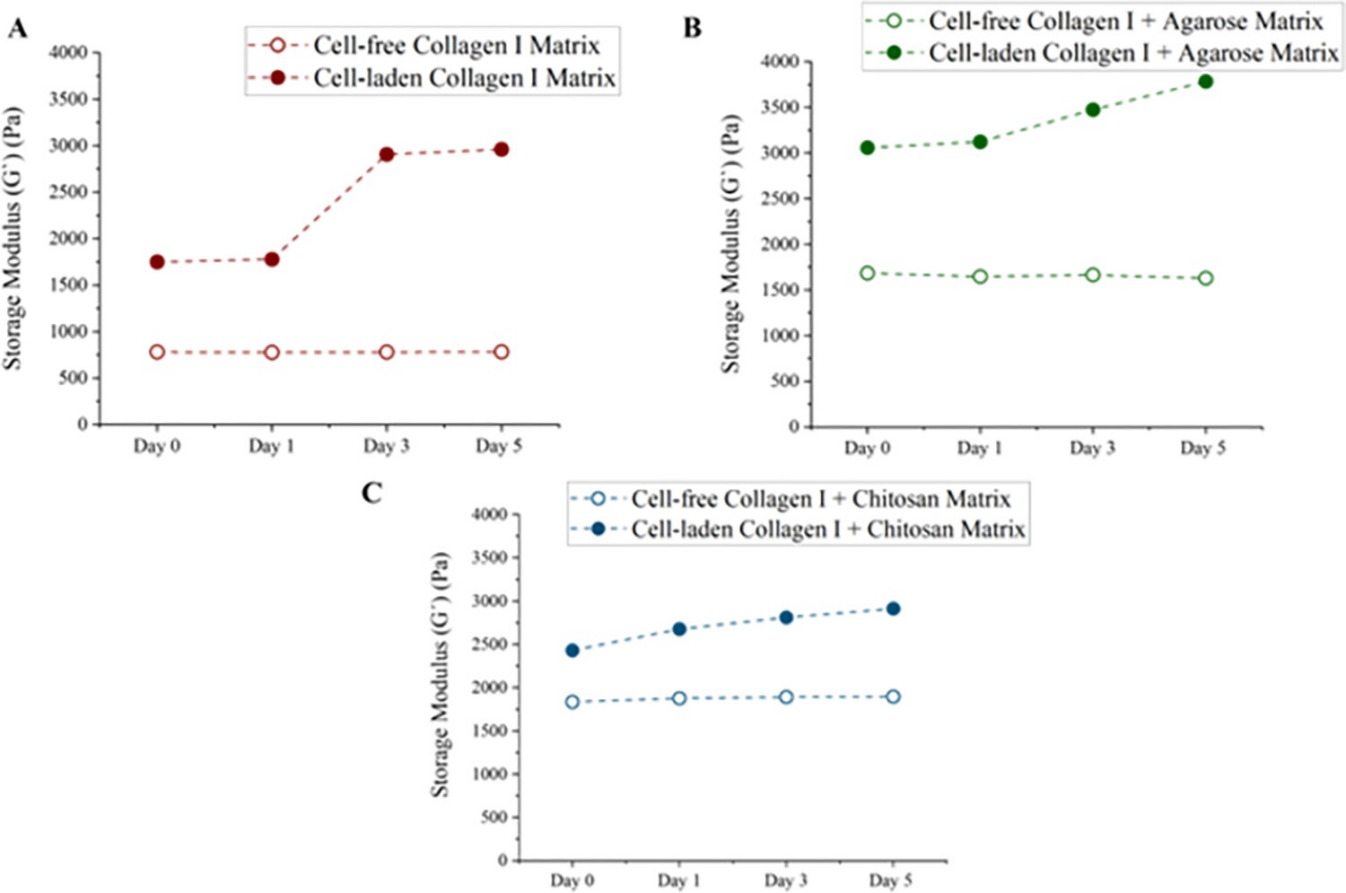
Rheological measurements of collagen I-based matrices. Storage modulus (G’) of 3 mg/mL collagen I only, 3 mg/mL collagen I supplemented with 2.5 mg/mL agarose, and 3 mg/mL collagen I supplemented with 10 mg/mL chitosan both with (red) and without (black) bone cells cultured for 5 days, are shown.

To understand the effects of chitosan and agarose on the stiffness of collagen I-based matrices, we determined the mechanical properties of the hydrogels with and without bone-related cells. More importantly, we utilized *in situ* contactless rheology which allowed monitoring of the ECM stiffness under cell culture conditions. The storage modulus (G´) gives information about the solid-like behavior of the hydrogels meaning that the higher the G´ value, the stiffer the gel. The storage moduli of the gels were determined for 5 days to ensure that the gels remained stable throughout the invasion and extravasation experiments. Simultaneously, it also allowed us to determine whether the presence of cells would have an impact on the stiffness. The average G´ values of the cell-free hydrogels were 779.91 ± 2.23 Pa, 1656.45 ± 20.71 Pa, and 1874.47 ± 24.11 Pa for collagen I only, collagen I + agarose and collagen I + chitosan hydrogels, respectively. The stiffness remained almost the same during the 5 days of measurement (Fig 3 and S1 Table). Importantly, we observed an increase in the G´ values of the gels for all the hydrogels in the presence of cells. The G´ value was increased from 1749.85 ± 53.46 Pa to 2961.35 ± 170.92 Pa in cell-laden collagen I only matrices, from 3058.39 ± 48.87 Pa to 3783.32 ± 35.16 Pa in cell-laden collagen I + agarose matrices and from 2430.58 ± 77.63 Pa to 2912.18 ± 42.90 Pa in cell-laden collagen I + chitosan matrices from Day 0 to Day 5 (Fig 3, S1 Table). Agarose-containing matrices displayed a higher stiffness compared to collagen I only and collagen I + chitosan matrices.

**Fig 4:**
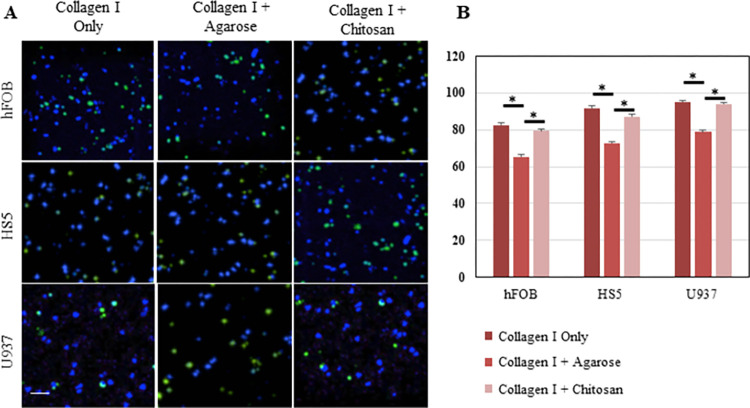
Viability of bone cell types in collagen I-based 3D matrices. (A) 3D merged images of hFOB, HS5, and U937 cell lines 3 days after they were cultured in hydrogels composed of collagen I only, collagen I with agarose and collagen I with chitosan. The green color represents dead cells, blue color shows all the nuclei. (Scale bar: 200 μm) (B) Percentage of viability for each condition is shown (n = 3). * p<0.01.

Next, we analyzed the viability of the cells in the selected hydrogels. In addition to hFOB osteoblasts and HS5 bone marrow stromal cells, we included U937 cells to represent monocytes, which are precursors of osteoclasts. Although adding agarose decreased viability for all cell types, 65 to 95% of the cells were viable in all conditions (Fig 4A and 4B). Thus, we focused on three hydrogel compositions for the subsequent experiments: collagen I only, collagen I supplemented with 2.5 mg/mL agarose (collagen I + agarose) and collagen I supplemented with 10 mg/mL chitosan (collagen I + chitosan).

### IC- and EX-chips provide a physiologically relevant bone metastasis model for breast cancer cells

**Fig 5:**
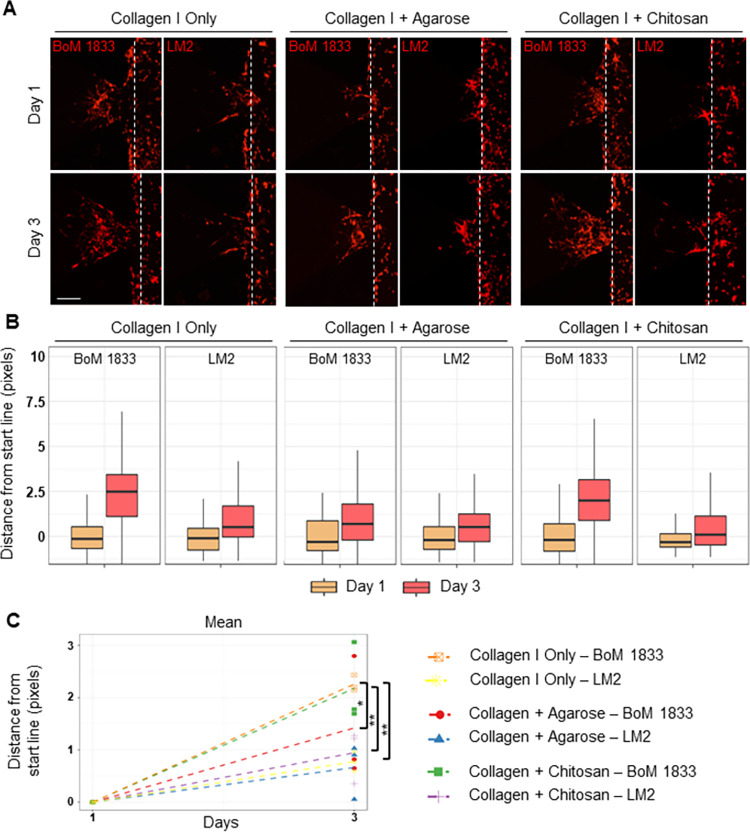
Invasion/chemotaxis of bone- and lung-specific metastatic clones of MDA-MB-231 breast cancer cells towards different bone-like microenvironments. (A) Representative Z-stack projection images showing invasion of bone-specific (BoM 1833) and lung-specific (LM2) clones of MDA-MB-231 breast cancer cells (red) towards HS5, hFOB, and U937-laden collagen I only, collagen I and agarose, or collagen I and chitosan hydrogels. Dashed lines represent the starting line of invasion/chemotaxis. (Scale bar: 100 μm) The distance of each bright pixel to the starting line was calculated and the data was normalized to day 1. (B) Distribution and (C) mean of distance values were plotted (n = 6). * p<0.01, ** p<0.005.

Finally, we investigated the invasion and extravasation potentials of MDA-MB-231 cells toward different bone-like microenvironment models. For this purpose, we used MDA-MB-231 cell line clones that specifically metastasize to bone (BoM 1833) and lung (LM2) tissues *in vivo* [20–22]. hFOB, HS5, and U937 cells were loaded in the HMC channel of IC-chip in collagen I only, collagen I + agarose, or collagen I + chitosan hydrogels. After 24 hours, bone-specific BoM 1833 or lung-specific LM2 clones were added into the MC1, and invasion was observed for three days (Fig 5A). BoM 1833 cells invaded longer distances than LM2 cells towards collagen I only and collagen I + chitosan hydrogels (Fig 5B). Although BoM 1833 cells also invaded collagen I + agarose hydrogel, the overall distances were shorter (Fig 5B). Furthermore, the mean distances were significantly lower compared to collagen I only and collagen I + chitosan conditions (Fig 5C, red compared to green and orange). Furthermore, the mean distances invaded were significantly higher for BoM 1833 compared to LM2 towards collagen I only and collagen I + chitosan hydrogels (Fig 5C orange, and green compared to yellow and purple, respectively).

**Fig 6:**
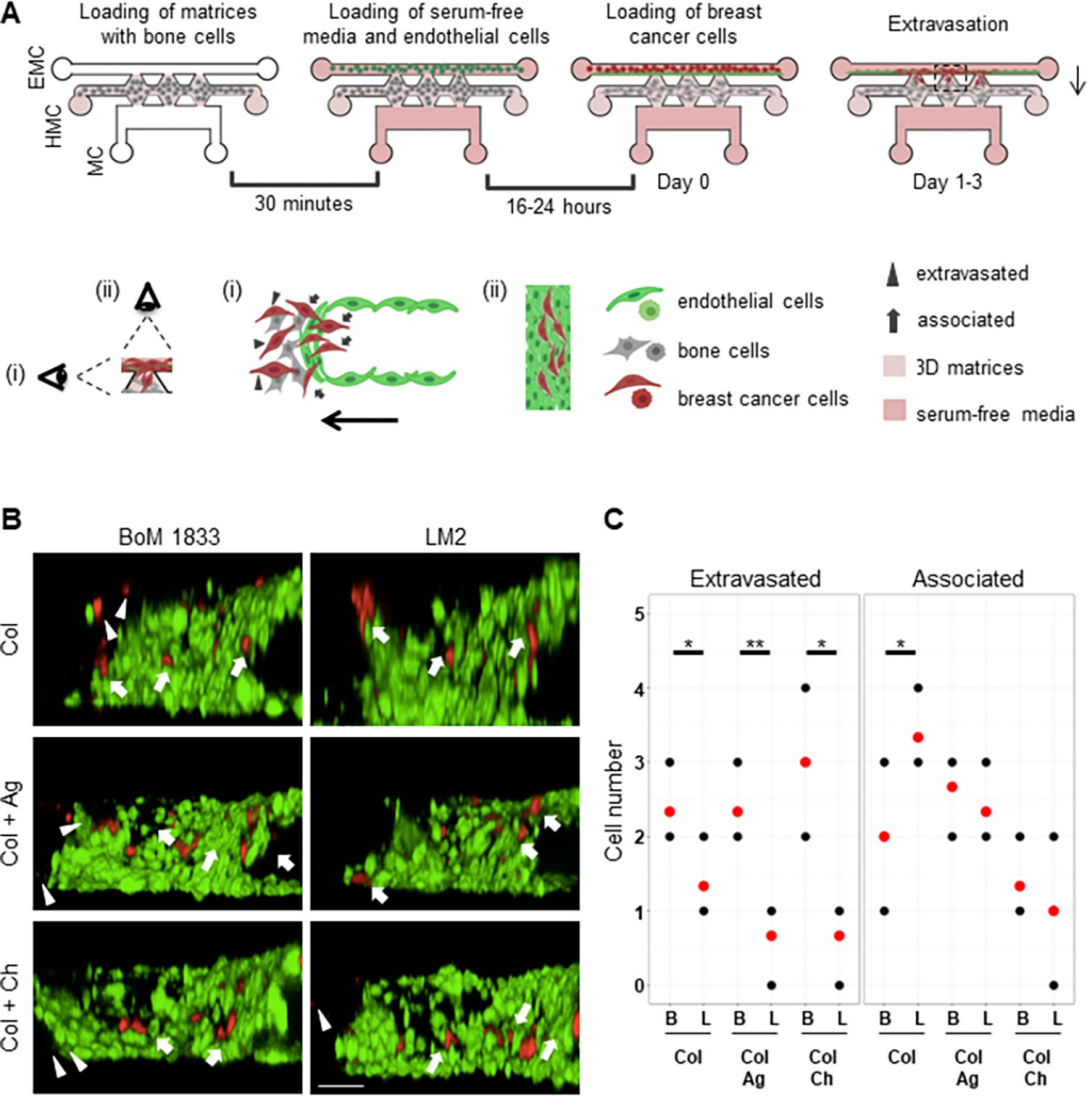
Extravasation of bone- and lung-specific metastatic clones of MDA-MB-231 breast cancer cells towards different bone-like microenvironments. (A) A schematic representation of the extravasation assay on EX-Chip is shown. On the bottom, a zoom-in inset of the imaging area between two posts, side (i) and back (ii) point of view of the endothelial layer (green) and extravasating cancer cells (red) are shown. Arrows indicate the direction of extravasation. (EMC: Endothelial Monolayer Channel, HMC: Homing Matrix Channel, MC: Medium Channel) (B) Representative 3D images showing extravasation of bone-specific (BoM 1833) and lung-specific (LM2) clones of MDA-MB-231 breast cancer cells (red) through HUVEC-C endothelial layer (green) towards HS5, hFOB and U937-laden collagen I only (Col), collagen I and agarose (Col + Ag), or collagen I and chitosan (Col + Ch) hydrogels. Arrow heads show extravasated and arrows show associated cancer cells. (Scale bar: 100 μm) (C) Number of extravasated and associated bone-specific (B) and lung-specific (L) MDA-MB-231 cells are shown. Each black dot represents one experiment, and the red dots represent mean values. (n = 2) * p<0.01, ** p<0.005.

After the formation of varying hydrogel conditions in the HMC channel, an endothelial monolayer was formed in the EMC channel using HUVEC cells labeled with a green fluorescent dye (Fig 6A). Then, BoM 1833 or LM2 cells were added to the EMC channel, and extravasation was observed for three days (Fig 6A and S4 Fig). BoM 1833 cells passed through the endothelial layer and extravasated toward all microenvironment models (Fig 6B). The number of extravasated cells was significantly higher for BoM 1833 than LM2 cells for all conditions (Fig 6C). LM2 cells preferred to stay associated with the endothelial layer failing to extravasate in collagen I condition (Fig 6C). Taken together, our data demonstrated that IC- and EX-chip platforms with 3D culture models composed of hFOB, HS5, and U937 cells embedded in collagen I only or collagen I + chitosan hydrogels represent a physiologically relevant bone-like microenvironment, on which invasion and extravasation behaviors of cancer cells correlate with their *in vivo* bone metastatic capacity.

Overall, our results demonstrate a novel bone-like microenvironment model on two lab-on-chip platforms, IC- and EX-chip, which can quantitatively demonstrate the invasion and extravasation potentials of breast cancer cells. Notably, the observed invasion and extravasation in LoC platforms correlate with their *in vivo* bone metastasis capacities. We tested the physiological relevance of the platforms by using two clones of the MDA-MB-231 breast cancer cell line, BoM1833, and LM2, which specifically metastasize respectively to bone or the lung *in vivo* [20–22]. We showed that BoM1833 invaded and extravasated towards bone-like microenvironment significantly more than LM2 on IC- and EX-chips, respectively.

The bone microenvironment consists of various cell types, including osteoblasts, osteoclasts, mesenchymal stem cells, immune cells, and stromal cells, constituting bone homeostasis together with the mechanical stimuli determined by stiffness and topography [23]. Here, we included human-derived bone marrow stromal cell (HS5), osteoblast (hFOB), and monocyte (U937) cell lines to partly mimic the cellular complexity of the bone microenvironment. Unlike the lung microenvironment, which was modeled by normal lung fibroblast cell line WI38 embedded in Matrigel [19] the bone microenvironment required several cell types to mimic the physiological condition. Mesenchymal stem cells (MSC), which were either left unstimulated [15, 17] or cultured under osteogenic conditions, [13, 14, 16] were previously used to model bone microenvironment. In our platform, co-culturing unstimulated mouse bone-marrow-derived MSC, D1, with bone marrow stromal cell and osteoblast cell lines did not induce further invasion of MDA-MB-231 cells. Since it requires 2 to 3 weeks of culture before the invasion or extravasation analysis can be performed, we did not induce differentiation of MSC in osteogenic medium to have a more straightforward method. Furthermore, with our approach, the microenvironment solely relies on the paracrine factors released from the resident cells. Thus, the behavior of cancer cells is not affected by special medium components, which might result in deviation from the physiological environment.

U937 cells can serve as an osteoclast precursor and can differentiate into osteoclasts upon stimulation with receptor activator of nuclear factor kappa-Β ligand (RANKL). Although we co-culture U937 cells with hFOB osteoblasts, which have RANKL expression, [41] we did not evaluate whether U937 cells differentiated. Thus, the absence of differentiated osteoclasts is one of the limitations of our model. Osteoclast activation results in bone resorption and the release of tumor-promoting factors, which will induce tumor cell proliferation and further trigger more osteoclast activation [42]. Osteoclasts are essential players in osteolytic bone metastasis and are involved in the regulation of dormancy [43]. However, their role in the homing of tumor cells to bone is not well established. Further studies incorporating differentiated osteoclasts in our model will allow the investigation of their contribution to the invasion and extravasation of breast cancer cells to bone.

The extracellular matrix is a crucial component of the tissue microenvironment, and it is involved in the regulation of cellular processes such as survival, proliferation, differentiation, and motility. Nearly 90% of the organic content of bone extracellular matrix is composed of collagen I [44]. Thus, we choose collagen I as the main matrix in our model, following previously proposed bone-on-chip models [12, 13, 18]. Collagen I alone does not represent the organic component of the bone microenvironment. Furthermore, stiffness is an important mechanical property of bone extracellular matrix. However, it is challenging to tune the mechanical properties of collagen I hydrogels. Therefore, we tested collagen I with other readily available biopolymers such as chitosan and agarose to tune the mechanical properties of the hydrogels. Chitosan is known to support the attachment of osteoblasts and the formation of mineralized matrix [37]. Agarose is bioinert and strain-stiffening properties at low concentrations [38]. The cells did not tolerate agarose concentration above 2.5 mg/mL, while chitosan was compatible with the cell viability and morphology. Bone-metastatic cells invaded the least towards agarose-containing hydrogel, while the invasion rate was similar towards collagen I only and collagen I-containing chitosan hydrogels. This observation is in line with a recent report showing that culturing on chitosan increased motility and stemness markers of cancer cells [45]. The difference between agarose- and chitosan-containing hydrogels could be due to the uncharged bioinert nature of the agarose, which contrasts with the positively charged chitosan. Agarose forms a fibrillar assembly and provides mechanical support to collagen I, but does not present adhesive sites, which affects gene expression profile and leads to different cellular phenotypes [39]. Although collagen I supplemented with agarose had increased the stiffness compared to the one supplemented with chitosan, this effect was not reflected on an increased invasive behavior. One reason could be the limited diffusion of the nutrients in the stiff gels to the cells which might lead to a more limited invasion of the cells toward that site. Both physical and chemical interactions between collagen I and the amino functional groups on chitosan might provide a more physiologically appropriate environment compared to agarose-based gels for the invasion of the cells. One factor that we did not investigate is the effect of different extracellular matrices on the proliferation of cancer cells. Thus, we cannot exclude the possibility that the invasion rates towards different hydrogel gels could be partly affected by the change in the cell numbers. Overall, it should be considered that the presence of agarose or chitosan could affect the behavior of a heterogeneous population of primary tumor cells based on their molecular profile.

In addition, several studies generated more stiff bone microenvironment models. Subcutaneous implantation of an insert containing collagen I and demineralized bone powder supplemented with Bone Morphogenic Protein-2 and -4 resulted in the generation of a mineralized bone marrow niche, which was able to populate hematopoietic stem and progenitor cells [7]. However, this approach requires eight weeks of *in vivo* incubation in mouse models, which would not be time and cost-efficient for future clinical applications. Another approach, which takes 14 to 30 days, is to allow pre-osteoblasts or MSCs differentiated under an osteogenic medium to secrete their extracellular matrix in the absence [10] or the presence of an artificial scaffold such as PolyHIPE [15]. Hydroxyapatite, which was shown to modify the mechanical properties of fibrin gel and induce angiogenesis [46] is another promising component to model bone extracellular matrix. Future studies are likely to assess the effect of hydroxyapatite.

Besides considering the importance of the target tissue microenvironment on breast cancer progression and metastasis, the factors residing in the primary tumor microenvironment (PTME) should be studied. Such studies may offer a detailed understanding of mechanisms and various factors playing key roles in the initiation of breast cancer bone metastasis. The progression of breast cancer is not only related to the genomic changes within cancer cells but also determined by the composition of the PTME. The crosstalk between breast cancer cells and stromal cells residing in the PTME has a major impact on the breast cancer progression, response to the treatments, and metastasis development as well as the survival of the breast cancer cells in PTME and target tissue microenvironments [47]. Therefore, a deeper understanding of the PTME is crucial for the development of personalized therapeutics for metastatic breast cancer.

Recently, it was shown that adipose-derived stem cells (ADSCs) give rise to multi-drug resistance of breast cancer cells by supporting cancer stem cells (CSCs) in breast PTME [48]. The cellular communication between ADSCs, and breast cancer cells results in the exchange of different intracellular components between these cells, leading to multi-drug resistance in breast cancer cells. Another study revealed the importance of breast PTME in identifying new therapeutic approaches by focusing on the spatial organization of the specialized cells within PTME and by reporting how different TME structures lead to apparent correlations with different breast cancer subtypes [49]. Our IC-chip and Ex-chip platforms can be easily used to model breast PTME and target tissue microenvironments to understand the complex crosstalk between the tumor and the microenvironment.

## Conclusion

Our bone-like microenvironment model generated on IC- and EX-chips by osteoblast, bone marrow stromal cell, and monocyte cell lines embedded in collagen I matrix was able to demonstrate the invasion and extravasation potential of MDA-MB-231 clones visually and quantitatively that correlate with their *in vivo* metastatic behavior. However, we should consider the possibility that primary breast cancer cells, which might have a varying degree of aggressiveness or are not accustomed to *in vitro* culture conditions, might require additional factors to present their in vivo behavior. In that scenario, our platforms might benefit from additional components, including different cell types such as osteoclasts, extracellular matrix secreted by resident cells, or additives such as bone powder or hydroxyapatite. Adapting our IC- and EX-chip models to test with primary tumor samples is essential to investigate the development of clinical tools for the prediction of metastatic risk and target tissue, as well as experimental tools to investigate molecular aspects of bone metastasis. Overall, the IC- and Ex-chips accommodating appropriate microenvironment models representing the *in vivo* behaviors of different cancer clones as promising platforms for the development of tools to predict the metastasis risk and the target tissue for breast cancer.

## Supporting information

S1 Fig Optimization of Collagen I concentration. HS5 cells were seeded in 2 and 3 mg/mL Collagen I hydrogels and visualized after 2 days in different Z planes (z1 –z4). White dashed lines represent the border of the gels. (Scale bar: 200 μm) (Distance between Z planes: 30 μm).
(TIF)

S2 Fig Cell morphology in hydrogels of Collagen I with or without chitosan. HS5 cells were cultured in 3 mg/ml Collagen I hydrogels without or with 10 mg/mL chitosan and visualized after 2 days. (Scale bar: 200 μm).
(TIF)

S3 Fig Cell morphology in hydrogels of Collagen I and agarose. HS5 cells were cultured in 3 mg/ml Collagen I hydrogels without agarose, or with 2.5 mg/mL, 5 mg/mL or 10 mg/mL agarose and visualized after 2 days. (Scale bar: 200 μm).
(TIF)

S4 Fig Representative images of breast cancer cells’ extravasation towards different bone microenvironments. Representative 3D images showing extravasation of bone-specific (BoM 1833) and lung-specific (LM2) MDA MB 231 cells (red) through the HUVEC-C endothelial layer (green) at Day 1 and Day 3. Bone microenvironments are formed by Collagen I only, Collagen I and agarose, or Collagen I and chitosan hydrogels with HS5, hFOB, and U937 cells. Rectangles represent the area shown in Fig 6A. (Scale bar: 200 μm).
(TIF)

S1 Table Rheology data obtained from each matrix condition. The average storage moduli (G´) together with the standard deviation values of different matrix compositions with and/or without bone cells from Day 0 to Day 5, is shown.
(TIF)
