# Supplementary figures and images for: Invasion/chemotaxis- and extravasation-chip models for breast cancer bone metastasis
Source: PLoS One. 2024 Oct 17;19(10):e0309285. doi: 10.1371/journal.pone.0309285 (PMC11486417; doi:10.1371/journal.pone.0309285)

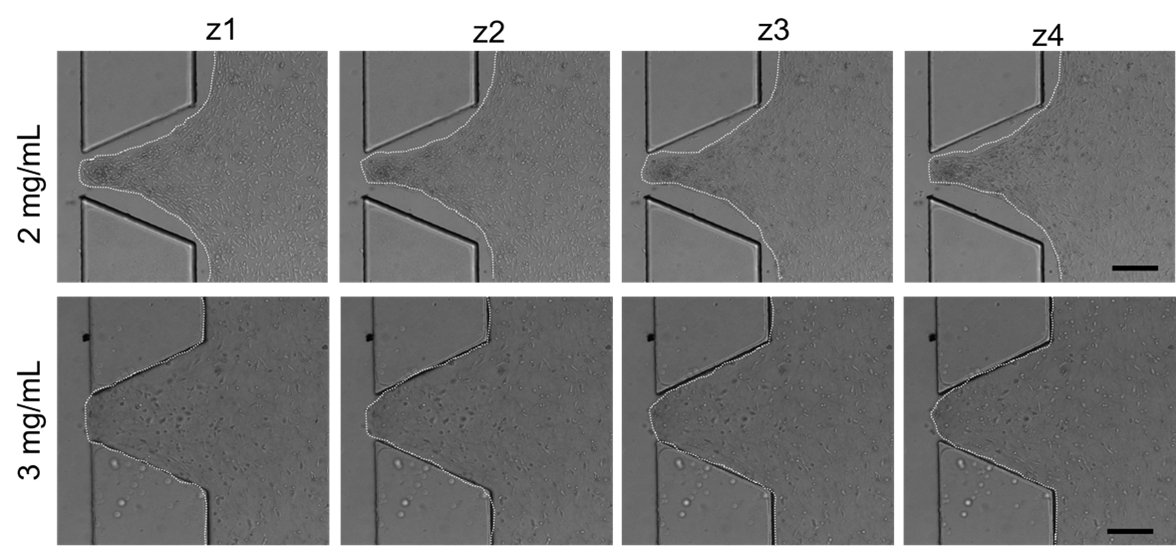

Supplement: S1 Fig — HS5 cells were seeded in 2 and 3 mg/mL Collagen I hydrogels and visualized after 2 days in different Z planes (z1 –z4). White dashed lines represent the border of the gels. (Scale bar: 200 μm) (Distance between Z planes: 30 μm). (TIF) [file pone.0309285.s001.tif]

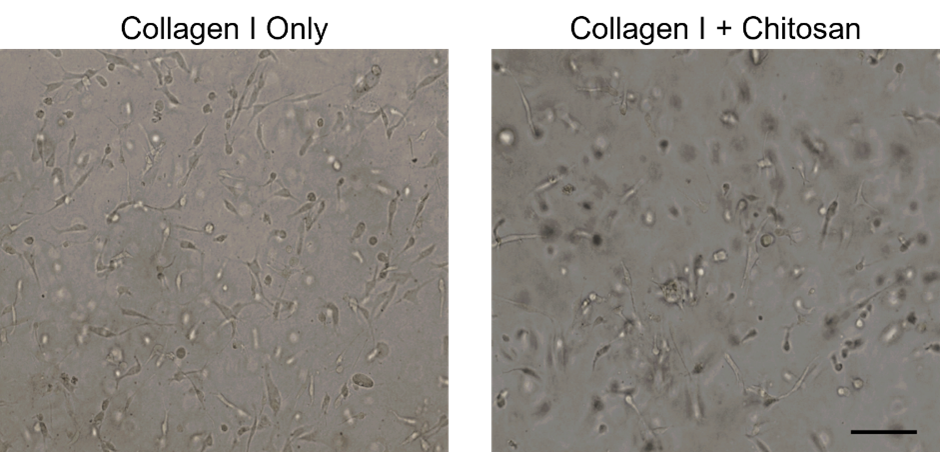

Supplement: S2 Fig — HS5 cells were cultured in 3 mg/ml Collagen I hydrogels without or with 10 mg/mL chitosan and visualized after 2 days. (Scale bar: 200 μm). (TIF) [file pone.0309285.s002.tif]

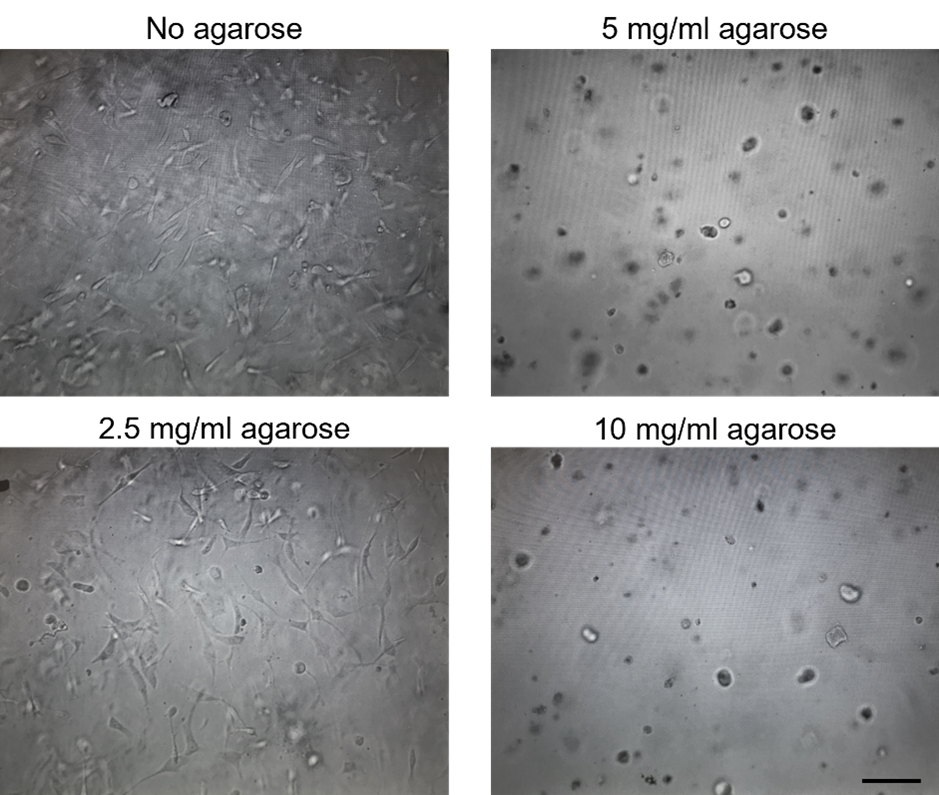

Supplement: S3 Fig — HS5 cells were cultured in 3 mg/ml Collagen I hydrogels without agarose, or with 2.5 mg/mL, 5 mg/mL or 10 mg/mL agarose and visualized after 2 days. (Scale bar: 200 μm). (TIF) [file pone.0309285.s003.tif]

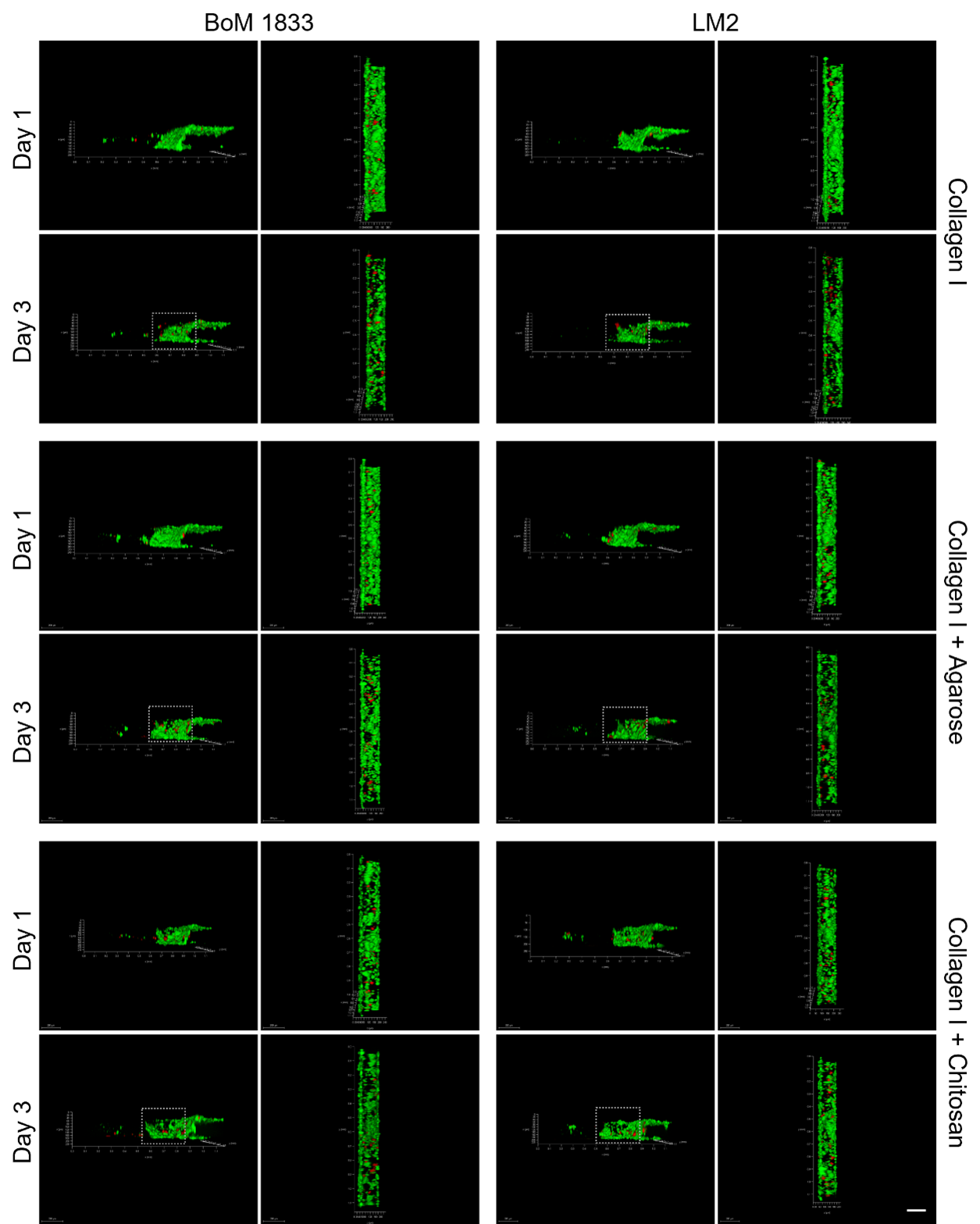

Supplement: S4 Fig — Representative 3D images showing extravasation of bone-specific (BoM 1833) and lung-specific (LM2) MDA MB 231 cells (red) through the HUVEC-C endothelial layer (green) at Day 1 and Day 3. Bone microenvironments are formed by Collagen I only, Collagen I and agarose, or Collagen I and chitosan hydrogels with HS5, hFOB, and U937 cells. Rectangles represent the area shown in Fig 6A. (Scale bar: 200 μm). (TIF) [file pone.0309285.s004.tif]

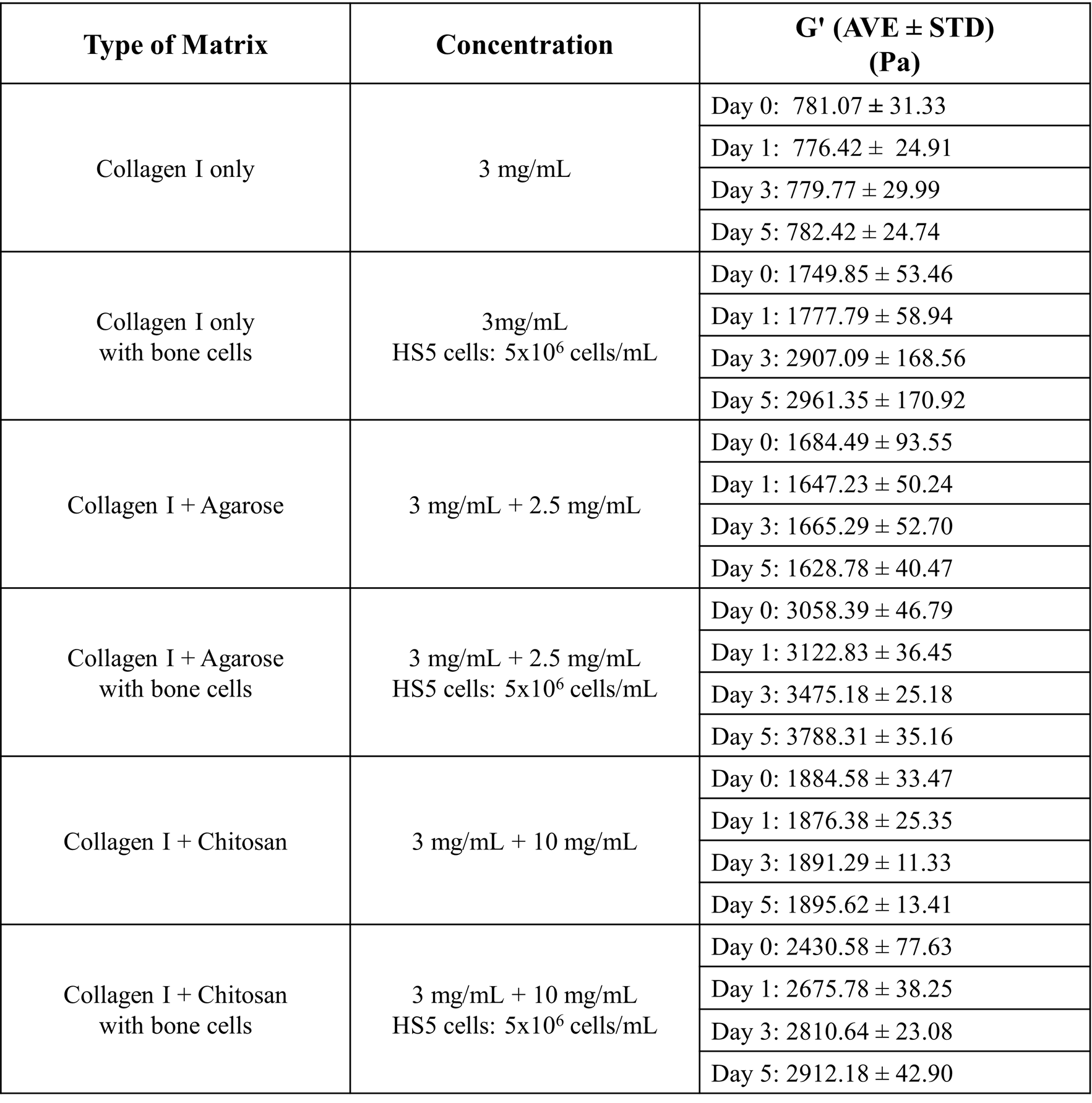

Supplement: S1 Table — The average storage moduli (G´) together with the standard deviation values of different matrix compositions with and/or without bone cells from Day 0 to Day 5, is shown. (TIF) [file pone.0309285.s005.tif]
